# Nonstructural protein 11 (nsp11) of porcine reproductive and respiratory syndrome virus (PRRSV) promotes PRRSV infection in MARC-145 cells

**DOI:** 10.1186/s12917-016-0717-5

**Published:** 2016-06-06

**Authors:** Xibao Shi, Xiaozhuan Zhang, Yongzhe Chang, Bo Jiang, Ruiguang Deng, Aiping Wang, Gaiping Zhang

**Affiliations:** College of Life Sciences, Henan Normal University, Xinxiang, 453007 China; Key Laboratory of Animal Immunology of the Ministry of Agriculture, Henan Provincial Key Laboratory of Animal Immunology, Henan Academy of Agricultural Sciences, Zhengzhou, Henan 450002 China; College of Veterinary Medicine and Animal Science, Henan Agricultural University, Zhengzhou, Henan 450002 China; Department of Bioengineering, Zhengzhou University, Zhengzhou, Henan 450000, 450002 China; Office of Science & Technology, Chongqing Police College, Chongqing, 401331 China

**Keywords:** PRRSV, Small interfering RNA, nsp11

## Abstract

**Background:**

Porcine reproductive and respiratory syndrome virus (PRRSV) induces one of most important devastating disease of swine worldwide, and the current methods poorly control it. Previous studies have indicated that the nonstructural protein 11 (nsp11) of PRRSV may be an important protein for the immune escape of PRRSV.

**Results:**

Here, we firstly explored the effect of over-expression of nsp11 on PRRSV infection and found that over-expression of nsp11 enhanced the PRRSV titers while the small interfering RNA (siRNAs) specifically targeting nsp11 could reduce the PRRSV titers in MARC-145 cells.

**Conclusion:**

In conclusion, PRRSV nsp11 promotes PRRSV infection in MARC-145 cells and siRNAs targeting nsp11 may be a potential therapeutic strategy to control PRRSV in future.

## Background

PRRSV, a positive sense and single-stranded RNA virus, is a member of family *Arteriviridae* [1]. Since it was emerged in the United States in 1987 and in Europe in 1990, PRRSV has rapidly spread in the swine producing regions and became one of the most important devastating diseases of swine worldwide. It can cause severe reproductive failure in sows and respiratory distress in young growing pigs [2]. Infection with PRRSV also made pigs easy to secondary infection by other pathogens [3]. Up to date, since there is no efficient method or drugs against PRRSV, it is very important and urgent to develop the effective therapeutic strategies to control PRRS.

The PRRSV genome has nine open reading frames (ORFs) composed of ORF1a, ORF1b, ORF2a, ORF2b, and ORF3-7. ORF1a and ORF1b could produce 16 nonstructural proteins (nsp1α, nsp1β, nsp2 etc.) [4–7]. Previous studies have shown that the nsp11 of equine arteritis virus(EAV), which is another member of family *Arteriviridae,* may play a key role in viral RNA synthesis and additional functions in the viral life cycle [8]. Other and our previous work also demonstrated that PRRSV nsp11 inhibited the host innate immune responses such as the transcription of type I interferon [7], the RNAi innate immune response [9] and the NLR family pyrin domain-containing 3 (NLRP3)-mediated production of IL-1β [10], which indicated that PRRSV nsp11 may play an important role in PRRSV infection. So the purpose of present study is to explore the effect of over-expression of nsp11 on PRRSV infection and whether the siRNAs targeting the PRRSV nsp11 could influence PRRSV infection.

## Methods

### Cell and virus

MARC-145 cells, derived from a monkey fibroblast cell line MA-104 [11], and 293T cells were grown in Dulbecco’s Modified Eagle medium (Gibco) plus 10 % heat-inactivated fetal bovine serum (Hyclone). PRRSV strain BJ-4 was a kind gift from Prof. Hanchun Yang (China Agricultural University).

### Plasmids

The pcDNA3.1-GFP-nsp11 plasmid was constructed by sub-cloning from the plasmid pcDNA3.1-FLAG-nsp11 [7] to pcDNA3.1-GFP [12] using the restriction endonuclease Hind III and EcoRI. The plasmids pcDNA3.1-FLAG, pcDNA3.1-FLAG-nsp11 and pcDNA3.1-FLAG-nsp11 H129A have been described in our previous work [7].

### Transfection of plasmids and viral infection

All newly-prepared plasmids were confirmed correctly by DNA sequencing. Transient transfection was carried out by using Lipofectamine 2000 (Invitrogen). Cells cultured in 24-well plates were transfected with the indicated expression plasmid or control vector (800 ng/well) in triplicate. And 6 h (h) later, the cells were infected with PRRSV at a multiplicity of infection (MOI) of 0.1, and then the cells were lysed by freezing and thawing three times after 24 h infection. The supernatants were collected by centrifugation, and the viral titers were detected by 50 % tissue culture infected dose (TCID50) assay using the method of Reed–Muench in the MARC-145 cells.

### Western blots

The 293 T cells were cultured in 24-well plates and transfected with pcDNA3.1-GFP-nsp11 or pcDNA3.1-GFP and nsp11 siRNA (100 nM) or control siRNA (100 nM) in triplicate, and 36 h later, the cells were lysed with lysing buffer (1 % Nonidet P-40, 0.1 % sodium deoxycholate, 0.1 % SDS, 50 mM Tris-HCl (pH 7.4), 150 mM NaCl, 2 mM EDTA, 2 mM Na3VO4, 2 mM NaF and a protease inhibitor cocktail). The detailed procedure for immunoblots has been described in our previous work [13]. Briefly, the proteins were separated by 10 % SDS-PAGE and transferred to polyvinylidene difluoride (PVDF) membranes (Millipore Company, Boston, Massachusetts, USA), and then the PVDF membranes were incubated with anti-GFP (Clontech) or anti-actin (Cell Signaling Technology) antibodies. Subsequently the membranes were incubated with appropriate secondary antibodies and were tested by an ECL detection system (Cell Signaling Technology, Boston, USA).

### Transfection of siRNA and viral infection

Cells cultured in 24-well plates were transfected with the indicated siRNA in triplicate (100 nM/well) (chemical synthesis by Bioneer) (Table. 1). And 6 h later, the cells were infected with PRRSV strain BJ-4 at an MOI of 0.1, and 24 h later, the cells were lysed by freezing and thawing three times. The supernatants were collected by centrifugation, and the viral titers were detected by TCID50 using the method of Reed–Muench in the MARC-145 cells.

**Table 1:** Synthesized siRNAs sequences targeting the regions of PRRSV nsp11 and the primers of ORF-7 for RT-PCR

Gene of target	Name of siRNA	Location(bp)	Sequence (5’-3’)
nsp 11	nsp11 siRNA 1	244-262	CGTGTCATACTATCTCACA
nsp 11	nsp11 siRNA 2	526-544	CACACTGACAGATGTGTAC
nsp 11	nsp11 siRNA 3	409-427	CACTACCGTTGGAGGATGT
Negative control	Control siRNA		CCTACGCCACCAATTTCGT
ORF-7 For			AAACCAGTCCAGAGGCAAGG
ORF-7 Rev			GCAAACTAAACTCCACAGTGTAA
GAPDH For			TGACAACAGCCTCAAGATCG
GAPDH Rev			GTCTTCTGGGTGGCAGTGAT

### Real time (RT)-PCR

MARC-145 cells cultured in 24-well plates were transfected in triplicate with nsp11-siRNAs/control siRNA and plasmid pcDNA3.1-GFP-nsp11 (800 ng/well) or pcDNA 3.1-GFP (800 ng/well). And 24 h later, the cells were infected with PRRSV at an MOI of 0.1, and 48 h later, the cellular RNA was extracted with TRIzol (Invitrogen). M-MLV reverse transcriptase was used for the PrimeScript™ RT Reagent Kit with gDNA Eraser (Takara, Dalian, China). Quantitative real-time RT-PCR (qRT-PCR) was performed using SYBR® Premix Ex Taq™ (Takara, Dalian, China) and was tested by the 7500 First real-time PCR system (Applied Biosystems, Foster City, CA, USA). Glyceraldehyde-3-phosphate dehydrogenase (GAPDH) was used as an internal control. The 2^−ΔΔCt^-method was used to analyze the relative amount of target gene expression.

### Viral titers

MARC-145 cells transfected with the nsp11 siRNA (100 nM) or control siRNA (100 nM) and the plasmid pcDNA3.1-GFP-nsp11 (800 ng/well) or pcDNA3.1-GFP (800 ng/well), and 24 h later, the cells were infected by PRRSV at an MOI of 0.1, the cells were frozen and thawed in three cycles and collected after 48 h infection. Then the viral titers were determined by TCID50 assay using the method of Reed–Muench in the cells of MARC-145.

### Statistical analysis

Statistical analyses were performed by Student’s t-test, and the differences were considered as statistical significance when *P* <0.05.

## Results

### Over-expression of nsp11 enhanced PRRSV titers in MARC-145 cells

**Fig. 1:**
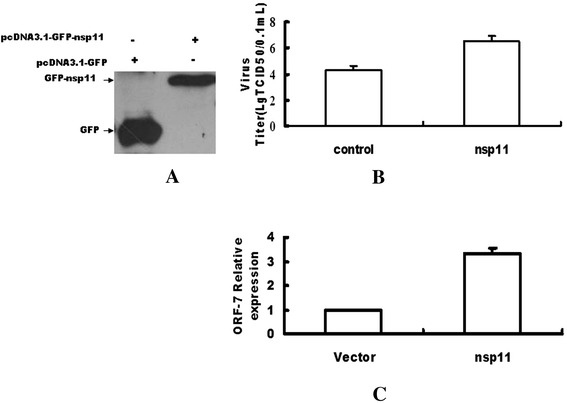
Over-expression of nsp11enhenced the titers of PRRSV. **a** 293T cells were transfected with pcDNA3.1-GFP (GFP) or pcDNA3.1-GFP-nsp11 (GFP-nsp11), and 48 h later, the cells were analyzed by western blots. **b** MARC-145 cells cultured in 24-well plate were transfected with pcDNA3.1-GFP-nsp11 (nsp11)(800 ng/well) or pcDNA 3.1-GFP (Con). And 6 h later, the cells were infected with PRRSV at an MOI of 0.1 or mock infected, and 24 h later, the cells were lysed by freezing and thawing three times in three cycles, then the viral titers were measured by TCID50. **c** MARC-145 cells cultured in 24-well plate were transfected with pcDNA 3.1-GFP-nsp11 (nsp11) (800 ng/well) or pcDNA3.1-GFP (Con). And 6 h later, the cells were infected with PRRSV at an MOI of 0.1 or mock infected, and 24 h later, the cells were collected and the viral RNA levels were measured by RT-PCR. Data represented means of three replicates, and experiments were repeated three times. Error bars represented the standard deviations. *: *P* <0.05 compared with the results in control. MOI: multiplicity of infection

Firstly, we successfully constructed the expression plasmid of pcDNA 3.1-GFP-nsp11 (GFP-nsp11) and the western blot in Fig. 1a confirmed the successful expression of GFP-nsp11 (Fig. 1). Secondly, the MARC-145 cells were cultured in 24-well plates overnight, and then the cells were transfected with the expression plasmid pcDNA3.1-GFP-nsp11 or the control plasmid pcDNA3.1-GFP. The results in Fig. 1b showed that the PRRSV titers from the MARC-145 cells transfected with pcDNA3.1-GFP-nsp11 were about one point six times to that from MARC-145 cells transfected with control plasmid, while the RNA levels of PRRSV from the MARC-145 cells transfected with pcDNA3.1-GFP-nsp11 were three times to that from MARC-145 cells transfected with control plasmid (Fig. 1c).

### siRNAs targeting nsp11 reduced PRRSV titers in MARC-145 cells

**Fig. 2:**
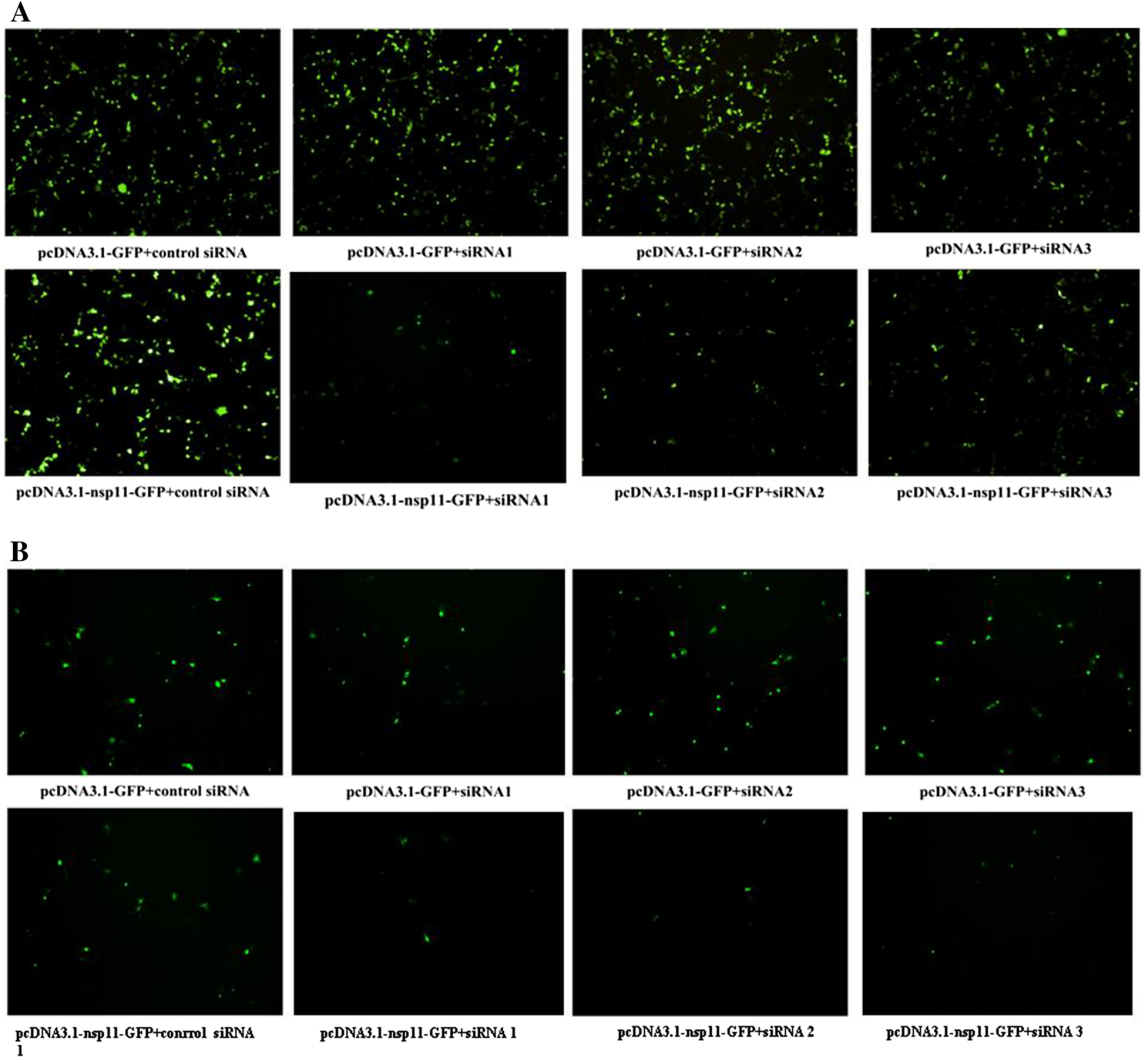
siRNAs targeting nsp11 could inhibit the expression of GFP- nsp11 and didn’t influence on the expression of GFP in 293T cells or in MARC-145 cells. 293T cells (**a**) or MARC-145 cells (**b**) cultured in 24-well plates were co-transfected with pcDNA 3.1-GFP-nsp11(800 ng/well) or pcDNA3.1-GFP (800 ng/well) and nsp11 siRNA 1 (100 nM), nsp11 siRNA 2 (100 nM), nsp11 siRNA 3(100 nM) or control siRNA (100 nM). And 24 h later, the cells were analyzed by fluorescence microscopy (50×). Data represented means of three replicates, and experiments were repeated three times

Now that over-expression of nsp11 could enhance PRRSV titers, it was reasonable to design siRNAs targeting nsp11 to investigate whether the siRNAs could reduce PRRSV titers. The sequences of the siRNA special for nsp11 and the control siRNA were listed in Table 1. 293T cells (Fig. 2a) or MARC-145 cells (Fig. 2b) grown in 24-well plates were co-transfected with the nsp11 siRNA (100 nM/well) or control siRNA and the plasmid GFP-nsp11 (800 ng/well). And 24 h later, the cells in five random fields were analyzed by fluorescence microscopy (50×) and only one of them was shown in Fig. 2. The results in Fig. 2 showed that all of the three siRNAs targeting nsp11 could inhibit the expression of GFP-nsp11 and didn’t influence on the expression of GFP.

**Fig. 3:**
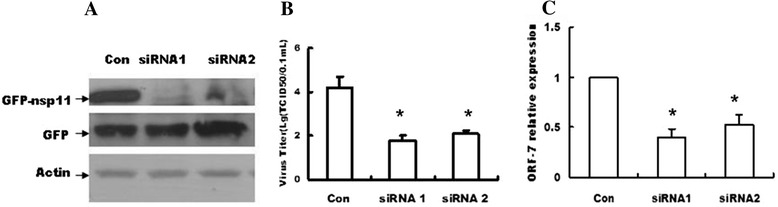
siRNAs targeting nsp11 could efficiently reduce the titers of PRRSV. **a** 293T cells grown in 24-well plates were transfected with pcDNA3.1-GFP-nsp11(800 ng/well) or pcDNA3.1-GFP (800 ng/well) and nsp11 siRNA 1 (100 nM), nsp11 siRNA 2 (100 nM) or control siRNA (100 nM), then 36 h later, the cells for collected for the western blot. **b** MARC-145 cells cultured in 24-well plates were transfected with nsp11 siRNA 1 (100 nM), nsp11 siRNA 2 (100 nM) or control siRNA (100 nM). And 6 h later, the cells were infected with PRRSV at an MOI of 0.1 or mock infected, and 24 h later, the cells were lysed by freezing and thawing three times, then the viral titers were measured by TCID50. **c** MARC-145 cells grown in 24-well plates were transfected with nsp11 siRNA 1 (100 nM), nsp11 siRNA 2 (100 nM) or control siRNA (100 nM). And 6 h later, the cells were infected with PRRSV at an MOI of 0.1 or mock infected, and 24 h later, the cells were collected and the viral RNA levels were measured by RT-PCR. Data represented means of three replicates, and experiments were repeated three times. *Error bars* represented the standard deviations. *: *P* <0.05 compared with the results in control. *MOI*, multiplicity of infection

Finally, we selected two siRNAs (siRNA1 and siRNA2) targeting nsp11 to determine whether siRNAs targeting nsp11 could reduce PRRSV titers in MARC-145 cell. The western blots results in Fig. 3a confirmed that siRNA1 and siRNA2 could efficiently reduce the nsp11 expression in 293T cells (Fig. 3a). The results in Fig. 3b and c showed that siRNAs targeting nucleic acid sequence of nsp11 could significantly reduce viral titers of PRRSV (Fig. 3b) and reduce RNA levels of PRRSV (Fig. 3c).

### The endoribonuclease activity of nsp11 was important for nsp11 to enhance the PRRSV titers

**Fig. 4:**
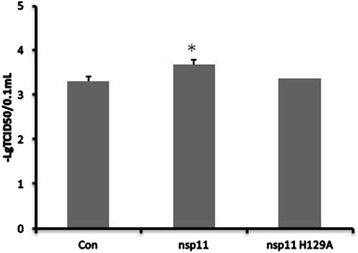
The endoribonuclease activity of nsp11 was important for nsp11 to enhance the PRRSV titers. MARC-145 cells cultured in 24-well plate were transfected with pcDNA3.1-FLAG-nsp11 (nsp11) (800 ng/well), pcDNA3.1-FLAG-nsp11 H129A (nsp11 H129A) (800 ng/well) or pcDNA 3.1-FLAG (Con). And 6 h later, the cells were infected with PRRSV at an MOI of 0.1 or mock infected, and 24 h later, the cells were lysed by freezing and thawing three times in three cycles, then the viral titers were measured by TCID50. Data represented means of three replicates, and experiments were repeated three times. *Error bars* represented the standard deviations. *: *P* <0.05 compared with the results in control. *MOI,* multiplicity of infection

Our previous work has shown that the endoribonuclease activity of nsp11 was important for nsp11 to inhibit the transcription of IFN-β [7] and the secretion of IL-1β [10]. So next we investigated whether the endoribonuclease activity of nsp11 was important for nsp11 to promote the PRRSV infection. Nedialkova et al. showed that His-129, His-144, and Lys-173 were the catalytic centers, and mutating one of the three amino acids could abolish its enzyme activity. The results in Fig. 4 showed that inactivating the endoribonuclease activity made nsp11 not promote the PRRSV infection.

## Discussion

nsp11 was a multi-functional protein. Both our and other previous studies have shown that PRRSV nsp11 is an interferon antagonist [7, 14] and that nsp11 plays an important role in viral RNA synthesis and in the viral life cycle [8]. Our recent work also demonstrated that PRRSV nsp11 inhibited the RNAi innate immune response [9] and NLRP3-mediated production of IL-1β [10]. While our present work showed that over expression of PRRSV nsp11 could enhance the titers of PRRSV (Fig. 1), so our present work gave a directly evidence that nsp11 was an important viral component for up-regulating the PRRSV titers.

Identification of and targeting viral important components is useful for developing viral vaccine and controlling the virus. For example, both the nonstructural protein 1 of influenza virus and the nonstructural protein 1 of mouse hepatitis virus (MHV) were important for the viral virulence respectively, and both the modified live-viral vaccines that deletion of nonstructural protein 1 resulted in complete protection against challenge with influenza virus infection and MHV infection respectively [15–17].

RNA interference (RNAi) is an exciting method to silence viral genes. Inhibition of specific genes by siRNA has proven to be a potential therapeutic strategy against viral infection [18], especially for the positive single stranded RNA viruses since their genomes function as both the mRNA and the replication template [19, 20]. Up to date, RNAi has been used against several viruses such as hepatitis B virus, foot-and-mouth disease virus, dengue virus and so on [20–22]. In this work, we also explore whether the siRNA, which targeted the nucleic acid sequence of nsp11, influenced the titers of PRRSV, and the results showed that siRNA targeting nsp11 significantly reduced the titers of PRRSV (Fig. 3). A recent improved live PRRSV vaccine has indicated that the ORF1a and ORF1b were the virulence determinants of PRRSV [23]. In addition, our recent work also show that RNAi innate immune response was an antiviral response to PRRSV and PRRSV inhibited this response by PRRSV nsp1α and nsp11, which indicated that targeting nsp11 would be useful for RNAi innate immunity against PRRSV [9]. So it is reasonable to propose that our present results gave a new clue for generating the new PRRSV vaccine by targeting PRRSV nsp11.

## Conclusion

In conclusion, our present study has shown that nsp11 was an important viral component for up-regulating the titers of PRRSV and that siRNAs directly targeting nsp11 could inhibit PRRSV infection, which indicated that PRRSV nsp11 may be an interesting target for controlling PRRSV in future.

## Abbreviations

EAV, equine arteritis virus; GAPDH, Glyceraldehyde-3-phosphate dehydrogenase; GFP-nsp11, pcDNA 3.1-GFP-nsp11; MHV, mouse hepatitis virus; MOI, multiplicity of infection; NLRP3, NLR family pyrin domain-containing 3; nsp11, nonstructural protein 11; ORF, open reading frame; PRRSV, porcine reproductive and respiratory syndrome virus ; PVDF, polyvinylidene difluoride; qRT-PCR, quantitative real-time RT-PCR; RNAi, RNA interference; siRNA, small interfering RNA; TCID50, 50 % tissue culture infected dose
